# An Address on Aseptic Midwifery

**Published:** 1899-09-23

**Authors:** Robert Jardine

**Affiliations:** Physician to the Hospital


### Hospital Clinics and Medical Progress.

AN ADDRESS ON ASEPTIC MIDWIFERY,
Delivered in the Glasgow Maternity Hospital, by Robert Jardine, M.D., &c., Physician to the Hospital.

(*Continued from page* 419.)

As the aseptic method of conducting labours is based on the facts which have been established by the study of the bacteriology of the vaginal secretions, I shall now give you a short epitome of the views of various observers.

The study of the bacteriology of the vagina dates from about 1887, when Gönner wrote his article on "The Micro-Organisms in the Secretions of the Female Genitalia in Pregnancy and Puerperal Diseases." Some work had been done before this, but the methods used had been faulty. He found on examining the secretions from 32 pregnant women that many organisms were present, but not pathogenic ones. He therefore concluded that auto-infection was impossible, and that antiseptic vaginal douches were unnecessary. In the same year Döderlein stated that he found the discharge from the uterine cavity perfectly sterile, but that that from the vagina, in 75 per cent., contained various micro-organisms, and not unfrequently pyogenic strepto- and staphylo-cocci. His conclusions were the very opposite to those of Gönner, viz., that auto-infection was possible and that vaginal douching was required. Since then many observations have been made, some confirming the view that the vagina is practically always aseptic, while others showed the very opposite. It would only weary you and serve no good purpose to take up all the different series of observations. In 1894 Krönig (after examining the secretions of 100 cases) came to the following conclusions,[1] viz., "That the vaginal secretions of pregnant women who have not been examined, no matter whether normal, pathological, or highly pathological, never contain organisms which grow aërobically upon the ordinary media at the body temperature, except yeast and gonococci, and therefore never contain septic bacteria. The vagina of every pregnant woman who has not been examined is therefore aseptic." He maintained that the pathogenic organisms found by other observers had been introduced into the vagina by their manipulations. He also stated that the vaginal secretions were always acid, but of a varying degree of acidity.

In the same year Krönig published an article upon the "Antibacterial action of the Vaginal secretions of Pregnant Women." He began a series of experiments to see what effect the vaginal secretions would have upon bacteria intentionally introduced into the vagina. He first introduced the innocuous bacillus pyocyaneus into the vagina of 20 pregnant women and found that it was destroyed in every case, the average time being 20 hours. He then experimented with staphylococci in 16 cases, and found they also disappeared in from 11 to 16½ hours. In three cases with streptococci they disappeared within six hours. He thus showed that the vaginal secretions possess a very powerful antibacterial or antiseptic action. He maintained that antiseptic douches, instead of destroying the pathogenic organisms which had been introduced into the vagina, only weakened or destroyed the normal antiseptic action of the secretions. It is probable that the acidity of the secretions, due to the action of the vaginal bacilli, have a good deal to do with this destruction of the pathogenic organisms, but he held there was also an antagonism between the vaginal and imported bacteria. The phagocytic action and the lack of oxygen also help in the battle.

It has been found by Menge, who confirmed Krönig's observations, that the vaginal secretions of non-pregnant women possess bactericidal action, but to a less marked degree.

Other observers have obtained different results from these. Last year Krönig and Menge published a work upon the "Bacteriology of the Female Genital Canal," giving the results of further investigations reaffirming their previous statements.

Krönig maintains that the observers who failed to find the vagina free from pyogenic organisms were using a faulty technique in obtaining the secretions. They used specula which could not be introduced without touching the edge of the hymen, while he used a small glass tube with an aspirator attached. It could be introduced without touching the edges of the hymen. Dr. Williams, of the Johns Hopkins University, who had in 1893 examined the secretions in 15 cases, and had found streptococci in 20 per cent., last year adopted Krönig's method of obtaining the secretions without touching any part of the hymen. In 92 cases observed he was able to confirm Krönig's observations. He therefore agrees with Krönig that the results of other observers are due to faulty technique.

The following are Dr. Williams' conclusions[2]: (1) We agree with Krönig that the vaginal secretions of pregnant women do not contain the usual pyogenic cocci, having found the staphylococcus epidermidis albus only twice in 92 cases, but never the streptococcus pyogenes or the staphylococcus aureus or albus. (2) The discrepancy in the results of the various investigators is due to the technique by which the secretion is obtained. (3) As the vagina does not contain pyogenic cocci, auto-infection with them is impossible; and when they are found in the puerperal uterus they have been introduced from without. (4) The gonococcus is occasionally found in the vaginal secretion, and during the puerperium may extend from the cervix into the uterus and tubes. (5) It is possible, but not yet demonstrated, in very rare instances, that the vagina may contain bacteria which may give rise to sapræmia and putrefactive endometritis by auto-infection. (6) Death from puerperal infection is always due to infection from without, and is usually due to neglect of aseptic precautions on the part of the physician and nurse. (7) Puerperal infection is to be avoided by limiting vaginal examinations as much as possible and cultivating external palpation. When vaginal examinations are to be made the external genitalia should be carefully cleansed and disinfected, and the hands rendered as aseptic as for a laparotomy. Vaginal douches are not necessary, and are probably harmful.

The act of parturition is a physiological one in normal cases, but it borders dangerously near the pathological, and very little may turn the scale. Every parturient woman is exposed to the danger of sepsis, but nature has provided for this danger by placing in the vagina certain organisms which possess the power of destroying the pathogenic ones which may enter, provided they are not too numerous. Over and above this you have the action of the phagocytes as in any other part of the body. Our endeavours should be to assist Nature in her beneficent action by preventing pathogenic organisms from gaining an entrance, and at the same time by not interfering with the benign organisms already present in the vagina. If pathogenic organisms have gained an entrance, we must endeavour to get them turned out as quickly and as thoroughly as possible. In all cases prevention is better than cure. Prevention can, I believe, be effected by a thorough system of asepsis, combined with the minimum of vaginal examinations. The important thing to remember is that the vagina of a pregnant woman is aseptic as a rule, and not only is it aseptic, but it possesses an antiseptic power capable of destroying in a few hours such virulent organisms as strepto- and staphylo-cocci. Give Nature a fair chance, do not handicap her, and she will do her work well. In my next lecture I shall deal with the practical side of this question, and shall endeavour to make plain to you how to conduct a labour aseptically.

[1] Amer. Jour. of Obstetrics, Oct., 1898. [2] Ibid.

